# Oxidative Stress, Cell Death, and Other Damage to Alveolar Epithelial Cells Induced by Cigarette Smoke

**DOI:** 10.1186/1617-9625-1-21

**Published:** 2003-09-15

**Authors:** K Aoshiba, A Nagai

**Affiliations:** 1 First Department of Medicine, Tokyo Women's Medical University, Tokyo, Japan

## Abstract

Cigarette smoking is a major risk factor in the development of various lung diseases, including pulmonary emphysema, pulmonary fibrosis, and lung cancer. The mechanisms of these diseases include alterations in alveolar epithelial cells, which are essential in the maintenance of normal alveolar architecture and function. Following cigarette smoking, alterations in alveolar epithelial cells induce an increase in epithelial permeability, a decrease in surfactant production, the inappropriate production of inflammatory cytokines and growth factors, and an increased risk of lung cancer. However, the most deleterious effect of cigarette smoke on alveolar epithelial cells is cell death, i.e., either apoptosis or necrosis depending on the magnitude of cigarette smoke exposure. Cell death induced by cigarette smoke exposure can largely be accounted for by an enhancement in oxidative stress. In fact, cigarette smoke contains and generates many reactive oxygen species that damage alveolar epithelial cells. Whether apoptosis and/or necrosis in alveolar epithelial cells is enhanced in healthy cigarette smokers is presently unclear. However, recent evidence indicates that the apoptosis of alveolar epithelial cells and alveolar endothelial cells is involved in the pathogenesis of pulmonary emphysema, an important cigarette smoke-induced lung disease characterized by the loss of alveolar structures. This review will discuss oxidative stress, cell death, and other damage to alveolar epithelial cells induced by cigarette smoke.

## Introduction

Cigarette smoking is a major risk factor in the development of lung diseases including, pulmonary emphysema, pulmonary fibrosis, and lung cancer. The mechanisms of these diseases include alterations in the alveolar epithelium, which is essential for the maintenance of normal alveolar architecture and function.

Alveolar epithelial cells are the lung's first line of defense against the external environment. These cells also produce surfactants to reduce surface tension, release cytokines to regulate inflammation, generates growth factors and matrix proteins to promote repair processes, and release proteinases and proteinase inhibitors to regulate the turnover of alveolar matrix proteins. When injured, alveolar epithelial cells participate in repair processes by initiating recruitment, proliferation, and differentiation into new alveolar epithelial cells. Therefore, alterations in alveolar epithelial cells may cause architectural and functional disruptions in the alveolar epithelium. In this context, exposure to cigarette smoke may induce an increase in epithelial permeability, a decrease in surfactant production, the inappropriate generation of inflammatory cytokines and growth factors, and an increased risk of lung cancer. However, the most deleterious effect of cigarette smoke on alveolar epithelial cells is cell death, i.e., either apoptosis or necrosis. Cell death induced by cigarette smoke exposure can largely be accounted for by an enhancement in oxidative stress.

Cigarette smoke contains more than 4,000 chemicals, many of which are known to be carcinogenic and injurious [1,2]. Importantly, cigarette smoke contains and generates various reactive oxygen species (ROS) and reactive nitrogen species (RNS), such as superoxide radical, hydrogen peroxide, hydroxyl radical, and peroxynitrite. Recent evidence indicates that the ROS and RNS in cigarette smoke damage alveolar epithelial cells. In this review, we will begin with a general description of alveolar epithelial cells and thereafter discuss oxidative stress, cell death, and other damage to alveolar epithelial cells induced by cigarette smoke.

## Alveolar Epithelial Cells

The alveolar epithelium is composed of two different cell types, alveolar type I cells and alveolar type II cells [3,4]. Alveolar type I cells are terminally differentiated, large, flat cells that line more than 90% of the alveolar surface and account for 7% of parenchymal lung cells [5]. They are relatively simple cells that contain a small nucleus, a few small mitochondria, some endoplasmic reticulum cisternae with ribosomes, interspersed microfilaments, and an inconspicuous Golgi apparatus, all located in the perinuclear cytoplasm [3]. The thin and attenuated cytoplasm of type I cells facilitates gas exchange by minimizing the diffusion distance between the alveolar gas and the blood.

Alveolar type II cells are predominantly located in the corners of the alveoli. They cover about 10% of the alveolar surface and account for 15% of all parenchymal cells [5]. Alveolar type II cells are cuboidal in shape and contain many organelles, including mitochondria, endoplasmic reticulum, microfilaments, and Golgi apparatus [4]. They also possess lamellar bodies, which are unique organelles that contain layers of surfactant phospholipids; surfactant proteins A, B, and C; lysosomes; and lysosomal enzymes [4]. The main function of alveolar type II cells is the synthesis and secretion of surfactant, a lipid and protein mixture involved in the reduction of surface tension in the alveoli. Alveolar type II cells are also involved in a variety of functions [4]. These functions include xenobiotic metabolism, through the activity of P450 enzymes [6]; the regulation of transepithelial ion transport [7]; the production of extracellular matrix proteins, such as fibronectin, type IV collagen, and proteogycan; and the expression of cytokines and growth factors, such as IL-6, IL-8, monocyte chemotactic protein-1, TNFα, TGFα, TGFβ, GM-CSF, and endothelin-1 [4]. Another important function of alveolar type II cells is their participation in epithelial repair processes. Following epithelial injury, alveolar type II cells migrate to the denuded area, proliferate into new alveolar type II cells, and differentiate into alveolar type I cells [4,8]. In this context, alveolar type II cells are considered to be the progenitors of alveolar epithelial cells.

## Systemic and Local Oxidative Stress Induced by Cigarette Smoke Exposure

Cigarette smoke has been calculated to contain 10^17 ^oxidant molecules/puff, of which 10^14 ^are ROS [1]. The gas phase of cigarette smoke mainly contains short-lived ROS, such as superoxide radical and nitrogen oxide, both of which immediately react to form highly reactive peroxynitrite. In contrast, the tar phase contains the long lived hydroquinones that undergo redox-cycle to form superoxide radical and hydrogen peroxide via semiquinones, thereby resulting in persistent oxidative stress [2,9,10]. Hydroquinones and hydrogen peroxide can enter cells and may even reach the nucleus, where they may cause oxidative DNA damage [9,11]. Some constituents of cigarette smoke may also release iron from ferritin, potentiating oxidative stress within lung cells [12]. Besides these direct mechanisms of enhanced oxidative stress, cigarette smoke also increases oxidative stress in the lungs by recruiting and activating phagocytes to release ROS [13]. Cigarette smoke also reduces extracellular and intracellular antioxidant capacity. For example, exposure to cigarette smoke decreases blood levels of antioxidants, including ascorbate, urate, ubiquinol-10, α-tocopherol, and β-carotene [14,15]. Acute cigarette smoke exposure also reduces glutathione levels with decreases in glutathione peroxidase and glucose-6 phosphate dehydrogenase activities in alveolar type II cells, erythrocytes, and lung epithelial lining fluids [16-20]. In contrast, chronic cigarette smoke exposure elevates glutathione levels with increases in those activities [19,21-23]. However, these adaptive increases in antioxidant levels may not be sufficient to protect the alveolar epithelial cells against oxidative stress in chronic smokers.

## Oxidative Damage in Alveolar Epithelial Cells from Cigarette Smoke Exposure

Cigarette smoke causes oxidative damage in alveolar epithelial cells. In vitro experiments with freshly isolated alveolar type II cells or the alveolar type II cell line (A549) have shown that cigarette smoke enhances intracellular ROS levels with a depletion of glutathione, thereby causing cell growth arrest, cell detachment, cell lysis, and enhanced epithelial permeability [18,20,24]. Importantly, cigarette smoke is also capable of activating alveolar and bronchial epithelial cells to elicit inflammatory responses. For example, volatile components of cigarette smoke, presumably acetaldehyde and acrolein, stimulate the release of IL-8 through the mechanism of protein kinase C activation [25-28]. Thus, cigarette smoke has either toxic or stimulatory effects on lung epithelial cells, probably depending on the magnitude of cigarette smoke exposure, the cell status, and the antioxidant levels of the cells.

Oxidative damage in alveolar epithelial cells by cigarette smoke includes oxidation of DNA adducts [29]. A previous study has shown that the acute exposure of mice to cigarette smoke induces oxidative DNA adduction, detected by an increase in 8-hydroxy-2'-deoxyguanosine (8-OHdG) levels in the heart, liver, and lung tissues [30]. Similarly, we have found that the immunohistochemical levels of 8-OHdG were dramatically increased in the alveolar and bronchial epithelial cells of mice one hour after cigarette smoke exposure; these levels then declined to their basal values after 16 hours [Aoshiba, unpublished observations]. Interestingly, we noted greater cellular levels of 8-OHdG in alveolar type II cells than in alveolar type I cells, suggesting that cigarette smoke imposes greater DNA damage in alveolar type II cells [Aoshiba, unpublished observations]. Our discovery of greater sensitivity of alveolar type II cells to cigarette smoke is interesting because alveolar type II cells are thought to be somewhat resistant to other inhaled oxidants such as NO_2 _and ozone [31]. We speculate that the sensitivity of alveolar epithelial cells to these gaseous oxidants may be different from their sensitivity to cigarette smoke, which contains many different oxidants in both gaseous and particle (tar) components. Alternatively, alveolar type II cells are more metabolically active than alveolar type I cells, and thus the basal levels of cellular oxidants may be higher in type II cells than in type I cells. Furthermore, type II cells are located at the corners of the alveolar septa, whereas type I cells reside between the corners, and this difference in location may influence the level of cigarette smoke exposure in the microenvironment. Alveolar type II cells are considered to be the progenitor of alveolar epithelial cells, and if type II cells are damaged by oxidative stress caused by repeated smoking, that may interfere with restoration of the alveolar epithelium. This process may be related to the alveolar destruction that leads to pulmonary emphysema in cigarette smokers.

Previous studies have indicated that hydroquinone and catechol are important constituents of cigarette smoke that initiate oxidative damage to the DNA [32,33]. For example, autoxidation of hydroquinone and catechol has been shown to generate hydrogen peroxide, which is iron-catalyzed to form the hydroxyl radical, leading to deoxyguanosine hydroxylation, endonuclease activation, and DNA strand breaks [32-37]. The hydroxyl radical also activates poly (ADP-ribose) polymerase (PARP), a 116-kd DNA repair enzyme that synthesizes poly (ADP-ribose) polymers in response to the formation of DNA strand breaks [38]. The activation of PARP causes a decrease in cellular nicotinamide adenine dinucleotide (NAD) levels that is sufficient to interfere with ATP synthesis and to cause cell death [39,40]. In fact, a previous study showed that the death of alveolar epithelial cells following cigarette smoke exposure was inhibited by the PARP inhibitor 3-aminobenzamide, supporting the involvement of PARP activation in cigarette smoke-mediated damage in alveolar epithelial cells [38]. In addition to ROS, reactive nitrogen species (RNS) may also contribute to the mechanism of DNA damage by cigarette smoke. For example, the exposure of bronchial epithelial cells to cigarette smoke produces xanthine and hypoxanthine, presumably resulting from the RNS-mediated deamination of guanine and adenine, respectively [41].

The exposure of lung cells to oxidative stress is generally followed by adaptive increases in the intracellular levels of many antioxidants including manganese superoxide dismutase (MnSOD), copper-zinc superoxide dismutase (CuSOD), catalase, and glutathione peroxidase (GP) [42]. Within cells, MnSOD is concentrated in the mitochondria. CuSOD and GP are mainly located in the cytoplasma and nucleus, and catalase is found predominantly in peroxisomes. The increased expression of these antioxidants protects cells from oxidative injury. An animal study has shown that rat exposed to cigarette smoke for 14 days exhibited an enhancement in the bronchial epithelial expression of antioxidant genes encoding manganese superoxide dismutase, glutathione peroxide dismutase, and metallothionein [43]. We also found that mice exposed to cigarette smoke for one hour exhibited an enhancement in the alveolar and bronchial epithelial expression of γ-glutamylcysteine synthetase (γ-GCS), the rate-limiting enzyme that catalyzes the de novo synthesis of glutathione (unpublished observations). Our in vivo findings were supported by in vitro experiments with A549 cells, which showed a dramatic depletion of intracellular glutathione, followed by the induction of γ-GCS gene expression in association with an increase in the DNA binding of AP-1/AP-1-like elements [19,20,24,44]. The expression of antioxidants such as γ-GCS and heme oxygenase-1 following oxidative stress is also known to be stimulated by the binding of the antioxidant response element to the Nrf2 transcription factor [45]. The adaptive synthesis of glutathione following cigarette smoke exposure may be relevant to the elevated glutathione levels in the epithelial lining fluid of cigarette smokers [19,21,46].

Few studies have examined the synthesis of anti-oxidants by alveolar type I cells compared to alveolar type II cells because the alveolar type I cells are difficult to culture in vitro. Several in vivo studies have shown that the cellular levels of glutathione-synthesizing enzymes, such as γ-glutamyltranspeptidase and glutathione peroxidase, are greater in alveolar type II cells than in alveolar type I cells [47,48]. The greater cellular levels of the antioxidant enzymes in alveolar type II cells than in alveolar type I cells may be important, because alveolar type II cells are more metabolically active than alveolar type I cells, and thus the basal levels of cellular oxidants may be higher in alveolar type II cells than in alveolar type I cells.

## Death of Alveolar Epithelial Cells Induced by Cigarette Smoke Exposure

Cigarette smoke-induced oxidative damages in alveolar epithelial cells ultimately causes cell death [20,49]. The type of cell death, either apoptosis or necrosis, depends on the magnitude of cigarette smoke exposure. A recent study with A549 cells has shown that aqueous extracts of cigarette smoke induced apoptosis at low concentrations (<5%) and necrosis at high concentrations (>10%) [49]. These cytotoxic effects were largely the results of ROS and aldehydes present in the volatile phase of cigarette smoke; the effects were attenuated by the oxidant scavenger N-acetylcysteine or the aldehyde scavenger aldehyde de-hydrogenase [49]. Interestingly, the cigarette smoke-induced apoptosis of A549 cells was not associated with the activation of caspases, which agrees with our findings on the cigarette smoke-induced apoptosis of alveolar macrophages [50]. These findings can be explained by the fact that activity of caspases requires cysteine residues, which is inactivated by the ROS in cigarette smoke [51]. Another study has shown that acrolein, a major volatile constituent of cigarette smoke, also induces the apoptosis of bronchial epithelial cells by generating ROS [52]. The in vitro ability of cigarette smoke to induce apoptosis was supported by an in vivo study demonstrating that the exposure of rats to cigarette smoke for approximately 100 days induced apoptosis of bronchial epithelial cells; the bronchial epithelial cells apoptosis was prevented by the oral administration of the antioxidant N-acetylcysteine [53]. Cigarette smoke exposure in rats has also been shown to augment asbestos-mediated DNA strand breaks and necrosis in bronchial epithelial cells [54].

The molecular mechanisms of the cigarette smoke-induced death of alveolar epithelial cells are not well understood. Recent studies have shown that hydrogen peroxide induces the apoptosis of alveolar or bronchial epithelial cells by activating a signaling pathway involving the depletion of glutathione, the activation of a Mg^2+^-dependent neutral sphingomyelinase, and the generation of ceramide [55-57]. However, whether cigarette smoke-induced apoptosis utilizes the same signaling pathway remains unclear because oxidative stress is capable of activating various signaling molecules, including NFκB, extracellular signal-regulated kinase ERK1/ERK2, and GADD genes [58,59]. On the other hand, cigarette smoke exposure may cause mitochondrial dysfunction, potentially leading to apoptosis or necrosis. For example, a recent study has shown that cigarette smoke exposure in mice causes mitochondrial DNA damage and protein nitration, with a reduction in mitochondrial enzyme activities in cardiovascular tissues [60]. Since the lung is the primary target of cigarette smoke exposure, cigarette smoke may also cause mitochondrial dysfunction in alveolar epithelial cells.

## Death of Alveolar Epithelial Cells in Cigarette Smoke-Induced Lung Diseases

Whether or not alveolar epithelial cells undergo cell death in healthy human smokers remains largely unconfirmed. However, recent evidence suggests that an enhancement in the death of alveolar epithelial cells may participate in the pathogenesis of pulmonary emphysema, an important cigarette smoke-associated lung disease characterized by the loss of alveolar cells. For example, clinical studies have recently shown elevated levels of apoptosis in epithelial and endothelial cells in the alveolar wall of smokers with pulmonary emphysema [61,62]. An animal study with rats has also shown that the chronic inhibition of the vascular endothelial growth factor receptor induced apoptosis in alveolar endothelial cells, followed by pulmonary emphysema [63]. Furthermore, we have found that alveolar epithelial transfection with active caspase-3 causes the apoptosis of alveolar epithelial cells and the subsequent development of emphysema in mice [64]. Taken together, these findings suggest that epithelial and/or endothelial apoptosis may contribute to the alveolar cell loss that characterizes pulmonary emphysema.

The loss of alveolar epithelial cells resulting from cigarette smoke exposure must be offset by the initiation of epithelial repair processes. However, cigarette smoke exposure has been shown to inhibit the ability of epithelial cells to migrate, attach to the extracellular matrix, and heal wounds [65,66]. Cigarette smoke exposure may also induce stress-induced senescence, preventing epithelial cell proliferation [Tsuji, unpublished observations]. Thus, cigarette smoke likely augments lung injury not only by inducing epithelial cell death, but also by inhibiting epithelial repair processes. These alterations in the alveolar epithelium following cigarette smoke exposure may contribute to the architectural and functional disruptions that cause the various lung diseases associated with cigarette smoking.

## Competing interests

The authors declare that they have no competing interests.
